# Cohesive vs. Non-Cohesive Gold

**Published:** 1880-08

**Authors:** W. E. Driscoll


### COHESIVE vs. NON-COHESIVE GOLD FOR FILLING TEETH.

*Read before the Indiana State Dental Society.*

BY W. E. DRISCOLL.

Mr. President, and Gentlemen of the Indiana State Dental Association:—Your Committee has designated myself as one of those they desired to have address you on the subject of filling teeth with non-cohesive gold; while others are named for the same duty on the subject of cohesive gold.

Believing that I could use the time much more profitably in giving my reasons for prefering cohesive to non-cohesive gold, I have done so rather than appear indifferent to the wish of your Committee when thus called upon. To come to the point then, I have deliberately discarded non-cohesive gold from my practice, and think I have done right, and can give sufficient reasons for the step.

First.—I know of no condition in which I can not make as good a filling with cohesive gold as I can with non-cohesive.

Second.—In a vast preponderance of cases I can make much better fillings with cohesive, than I can with non-cohesive gold. And it may not be out of place for me to say, that in the great majority of cases I believe I can make better fillings with cohesive gold, than anybody can make with non-cohesive gold.

I have no doubt that many can scarcely restrain their impatience in listening to what they consider so mistaken and pernicious teachings. I also recollect when I thought it best to use the two kinds discriminatly. But now I feel that the reason why I made that much concession in favor of non-cohesive work, was, because I had not progressed so far as I have since done in mastering the mysteries of cohesive gold work. And if I apply this criticism to myself I must be excused if I say I believe that if many of the strong advocates of non-cohesive gold, knew better how to manipulate cohesive gold they would not have much use for the non-cohesive.

But they claim a large list of advantages in favor of non-cohesive gold, which I will examine so far as I can at present recall them to mind.

First.—Its superior adaptability to the walls of cavities and especially to the frail edges of soft teeth. Now this may be true if we undertake to manipulate the cohesive gold in the same manner as is best calculated for the management of non-cohesive gold. But is that a fair test? Is it any more so than to try to fill with non-cohesive in the way best adapted to cohesive and then condemn it on such a test? We are all open to more reason than that.

Cohesive gold can be welded only within given thicknesses or bulk, and if the attempt is made to consolidate a larger fold or pellet than its nature will permit, then the work is defective, just as non-cohesive work would be if we tried to weld it, instead of wedging it according to its nature and capabilities. So, too, cohesive gold lacks the quality of kindly and easily taking the impression of an irregular form against which it is pressed, if too large a quantity is essayed to be forced into such shape at once. But this is entirely avoided by placing the proper amount of gold exactly where it is going to stay, and welding or impacting it there before additions are made. This plan is met with the objection that it is slower than the use of large bulks of non-cohesive gold. Properly considered there is nothing in this objection to effect our decision. If the cavity is a simple one and very deep and large, foundation cement may be used for much the larger bulk of the work and the tooth made all the more comfortable and safe by its use. If the cavity is compound and shallow the cohesive filling can be made as quickly as the non-cohesive can. So as I have said there is really no time saved by using non-cohesive gold, if our management of the case is as it should be.

As to the proper thickness of the layers of gold, I have, after using about everything that has been on the market the past fourteen years, some of them for years and some for months, settled down to No. 60 cohesive gold foil, for all kinds of fillings I have to make. I have remnants of a number of thicknesses of foils, blocks, pellets, non-cohesive and cohesive, but I feel entirely disinclined to work with them any more, except occasionally to demonstrate to a student or friend my theories in such work.

Very few, of course, will make as good fillings at the first trial of a new form of gold, as with that which has long been familiar. But I believe that with proper training, any one can learn to make better fillings with No. 60 cohesive gold foil than with any other form or quality of gold. And I do not believe it will require anything like the amount of time to learn to manage it expertly, that it takes to master the difficulties encountered with other forms.

It is urged against cohesive gold that in adapting it to the walls of cavities in soft teeth the edges are crumbled, and a defective filling is the result. I will say I have done my share of this kind of work, but it was my fault, not that of the gold. I have never seen a tooth so soft that it would crumble if the edges were properly rounded before commencing the insertion of the gold; and I examine all my work with a strong magnifier before discharging the case. Then it is urged that thin walls, show cracks in the enamel, after being filled with cohesive gold. That is true with any other number than No. 60 or practically the same thickness, but with this number any one will soon see that where any approach to such a result supervenes, it can be easily avoided by taking the proper precautions.

It is said so many cohesive gold fillings have failed, and some of them too, were made by operators of great reputation in the profession. Very well, great operators make poor fillings with any material sometimes; we all do it, and there are conditions when a tooth will decay, without we could seal it up entirely from the contact of the fluids of the mouth. Will any one bring before us a case of this kind, where non-cohesive gold has saved the teeth. If he can it will be the first indispensable step toward settling the question.

I do not deny that where the conditions are favorable a well trained hand in the use of non-cohesive gold, can make a very good filling. But I do say, that with proper training, every such case can be filled as well with cohesive as with non-cohesive gold. Then, as I said at first, there is a vast preponderance of cases in which non-cohesive work will bear no comparison to that made of cohesive gold. Then after these is an enormous number of cases in which non-cohesive gold can not be made to stay at all, but can be made almost as good as new with cohesive gold. If this is true, it would be better for the dentist in average practice, to confine himself to as near one form and quality of gold as possible. For it can not be truthfully denied that a large portion of the dentists of the country, do not get enough gold filling to do to keep them in good training, and while they should be encouraged to think and investigate, and experiment, it must be admitted that there is one best way to do almost everything, and I say, for general practice, the more who adopt No. 60 cohesive gold foil for filling teeth, the better it will be for all concerned.

